# Examining the relative influence of dispersal and competition on co-occurrence and functional trait patterns in response to disturbance

**DOI:** 10.1371/journal.pone.0275443

**Published:** 2022-10-07

**Authors:** Brandon S. Schamp, Riley Gridzak, Danielle A. Greco, Thomas Michael Lavender, Anusha Kunasingam, Joanna A. Murtha, Ashley M. Jensen, Aksel Pollari, Lidianne Santos

**Affiliations:** 1 Department of Biology, Algoma University, Sault Ste. Marie, Ontario, Canada; 2 Department of Biology, Queen’s University, Kingston, Ontario, Canada; Irstea, FRANCE

## Abstract

Disturbance gradients are particularly useful for understanding the relative influences of competition and dispersal. Shortly after disturbance, plant composition should be influenced more strongly by dispersal than competition; over time, this should reverse, with competition becoming more important. As such, we predicted that plant functional traits associated with high dispersal ability would be over-represented shortly after a disturbance event occurs, while those associated with high competitive ability would have increased representation as time progresses. Additionally, it has been suggested that competitive interactions may contribute to negative co-occurrence patterns; if this is the case, negative co-occurrence patterns should also increase as time-since-disturbance increases. Here, we examine how functional trait and co-occurrence patterns change over time following a herbicide-based disturbance, compared to undisturbed vegetation, in a temperate, old-field grassland dominated by herbaceous perennials. In our study system, negative co-occurrence patterns were most pronounced in disturbed plots one year after herbicide application, consistent with several lines of evidence that dispersal can strongly impact both composition and co-occurrence patterns. Over three years post-disturbance, co-occurrence patterns in disturbed plots decreased, becoming more similar to control plots. This pattern is inconsistent with the expectation that competition contributes to negative co-occurrence patterns, at least over three growing seasons. More pronounced negative co-occurrence patterns were associated with higher species evenness among plots. Functional traits related to increased dispersal (mean seed mass, and proportion of stoloniferous/rhizomatous species) and competitive ability (mean species height, and mean specific leaf area) did not differ significantly across treatments, with the exception of mean height in the third-year post-disturbance; however, the overall trajectory of this trait was inconsistent with theoretical expectations. Overall, co-occurrence patterns changed across the gradient of time-since disturbance, but not as expected; functional trait patterns (trait means, functional diversity measures) were not responsive to our experimental disturbance gradient.

## Introduction

Species co-occurrence patterns reflect the tendency for some species to consistently be found growing together (positively co-occurring), or apart (negatively co-occurring). Negative co-occurrence patterns are fairly common in natural systems (e.g., [1,2]), and are of particular interest because they may represent, at least in part, a signature of antagonistic interactions among species (i.e., competition) [3,4]. Hypothetically, in a scenario where two competing species grow close together, the weaker competitor is excluded, such that at the community-scale, they present a pattern of negative co-occurrence. Intransitive competition (i.e., non-hierarchical competition) among species may also contribute to negative co-occurrence patterns [5]. There is considerable evidence that competition plays a role in natural plant communities [6–8]; however, there is little evidence that competitive interactions are an important driver of negative co-occurrence patterns ([9], but see [10]).

While competition’s role in co-occurrence patterns has been of great interest, other factors can influence these patterns. Environmental filtering [11] can contribute to patterns of co-occurrence if a focal community spans important abiotic gradients (e.g., precipitation or temperature [12,13]) to which constituent species are differentially adapted [2,4,14]. For example, if a field contains a soil moisture gradient, samples at one end of the gradient may contain species more tolerant of wet conditions, while samples at the other end may contain species adapted to drier conditions. Species in sample plots spanning this gradient, for example, are more likely to negatively co-occur. Other processes, such as dispersal–which we discuss in more detail below–can also impact co-occurrence patterns (e.g., [9,10,15–19]). This body of research makes it clear that species co-occurrence patterns within natural systems can be the product of multiple processes. Consequently, it is not reasonable to draw conclusions about processes driving these patterns using only a null model test that reveals non-random co-occurrence patterns [20]. To understand the processes governing these patterns, null models must be combined with experimental and/or analytical approaches that can clarify the relative contribution of specific processes to species co-occurrence patterns observed in nature [20]. If the goal is to understand the contribution of competition to co-occurrence patterns, the influence of factors such as dispersal or abiotic variation must either be controlled experimentally or integrated into analyses.

Dispersal can contribute to patterns of species co-occurrence in several ways. For example, Ulrich [15] and Bell [16] both demonstrated that negative co-occurrence patterns might arise from neutral dynamics, with dispersal playing a prominent role. Dispersal can allow weaker competitors to persist at a particular site, or continually deliver seeds to areas where that species is poorly adapted to local abiotic conditions. In this way, dispersal can contribute to co-occurrence patterns through mass effects [21]. At least one experimental study has demonstrated that a system’s dispersal regime can profoundly impact co-occurrence patterns [19]. At larger scales, some species may not be able to reach some sites because of differences in dispersal ability. Such dispersal limitations among species can contribute to negative co-occurrence patterns, even when all species involved are adapted to live in all abiotic conditions under consideration [22]. While dispersal can affect co-occurrence patterns at all scales, it is possible to reduce the influence of dispersal limitation on these patterns in the same way that it is possible to reduce the influence of abiotic gradients–by conducting research in smaller scale, relatively homogeneous communities (e.g., [9,10]).

The influence of dispersal on species co-occurrence patterns is of particular interest in light of broader efforts to understand the relative importance of dispersal and competition in structuring natural communities (e.g., [10,23–26]). Importantly, these two mechanisms represent ends of a gradient characterized by different levels of disturbance. When a site is recently or frequently disturbed, the supply of resources will generally exceed demand [27]. Under these conditions, competition is necessarily less important [28–31] and disturbance-based mortality opens up spaces and resources that must be captured by dispersal. Conversely, when a site is infrequently disturbed, or in the late stages of post-disturbance succession, competition should play a more prominent role in driving community composition, while dispersal’s role should be reduced [32]. Accordingly, evaluations of co-occurrence along gradients of time-since-disturbance can inform us about the relative roles of dispersal and competition in governing species co-occurrence patterns [10]. It is increasingly recognized that examining changes in co-occurrence patterns over time [9,10] and across experimental treatments [20], represents a valuable avenue for pursuing greater understanding of the mechanisms underpinning species co-occurrence patterns.

Evidence indicates that in densely growing herbaceous vegetation, disturbance and mortality are primary sources of gaps that local dispersal fills [33,34]. Gap creation is especially important for diversity maintenance in grasslands [35], as many species are microsite [36,37], and seed limited [38]. When disturbances are pronounced and/or frequent, composition in samples/plots is governed by which species disperse into those gaps as seeds (either in seed rain or from seed banks) or by clonal spread from organs such as rhizomes or stolons. In the present experiment, the use of glyphosate to disturb vegetation may have reduced the likelihood of colonization from seed banks [39] and/or bud banks [40]; thus, increasing the importance of dispersal from seed rain and clonal spread from further away. Likewise, when disturbance is reduced and/or infrequent, dispersal into gaps is less important, and a plant species’ ability to successfully compete in dense neighbourhoods will determine what is found growing in a given sample/plot. Our research extends from what research on succession indicates about the changing influence of competition along successional gradients [32], and from previous research that has explored changes in co-occurrence along an early successional gradient [10].

Species interact with their environment and each other via their phenotypic characteristics. Consequently, ecology has naturally progressed towards efforts to understand ecological patterns using functional traits [41]. For example, evidence from competition studies suggests that greater maximum height gives plants a competitive advantage (e.g., [42–46]). Other traits, like specific leaf area, may also contribute to a plant species’ competitive ability [44]. Seed mass is an important functional trait when considering dispersal and establishment (e.g., [47,48]). Seed mass is often associated with a competition-colonization trade-off, where species allocate resources to increase either their competition or colonization ability, but not both [49,50]. For example, possessing a large seed mass aids in establishment success of seedlings post-disturbance [51] but results in reduced fecundity of the parent plant [52,53]. Finally, the tendency to spread into gaps via clonality may represent an important trait for species responding to recent disturbance. Longer distance dispersal via stolons or rhizomes may allow established plants to quickly capture space vacated by disturbance [54]. Consequently, there is clear reason to test whether patterns of species height, specific leaf area, seed mass, and the proportion of rhizomatous or stoloniferous species change along a gradient of time-since-disturbance. Combining co-occurrence and functional trait patterns is increasingly recognized as a powerful method for examining community assembly processes [55,56].

In this study, we set out to understand the relative impacts of dispersal and competition on co-occurrence and functional trait means and diversity measures (dispersal: seed mass, proportion of stoloniferous/rhizomatous species; competition: species height, specific leaf area) in an old-field plant community. We tested how these patterns changed over three years following an experimental disturbance treatment that killed approximately one-third of resident plants in half of our plots. A considerable body of evidence has demonstrated that disturbances, particularly those that induce mortality, can significantly influence community structure, supporting the persistence of some early successional species (e.g., [57,58]). A gradient of time-since-disturbance acts as a gradient of the relative influence of dispersal and competition. Based on this, we predicted an increase in species richness, evenness, and dissimilarity in disturbed plots compared to control plots; we predicted that these measures would be highest in the first year following the disturbance treatment due to successful gap colonization, and decrease thereafter as the role of competition becomes more important. We tested whether co-occurrence and functional trait patterns (trait means, and functional diversity measures) changed directionally along this gradient [10], to better understand how these patterns are governed, or not governed, by dispersal and competition.

## Methods

### Study site

The study site is an old-field plant community within the Ontario Forestry Research Institute’s Arboretum in Sault Ste. Marie, Ontario, Canada (46.53° N, 84.45° W). This community has been undisturbed, excluding treatments, since 2007, before which it was mown once per year. Vegetation at this site consists of 40 herbaceous perennial plant species (70% forbs and 30% graminoids), with occasional shrub species. The growing season at our old-field plant community spans from May to October. Peak flowering occurs in July, with few species flowering early and late in the season [59]. Several plant species within our study community are non-native, but have long been naturalized in this region and are common in fields (S1 Table). Examples of common species include *Potentilla recta*, *Phleum pratense*, *Carex gracillima*, *Lotus corniculatus*, and *Phalaris arundinacea*.

### Treatment plots

**Fig 1:**
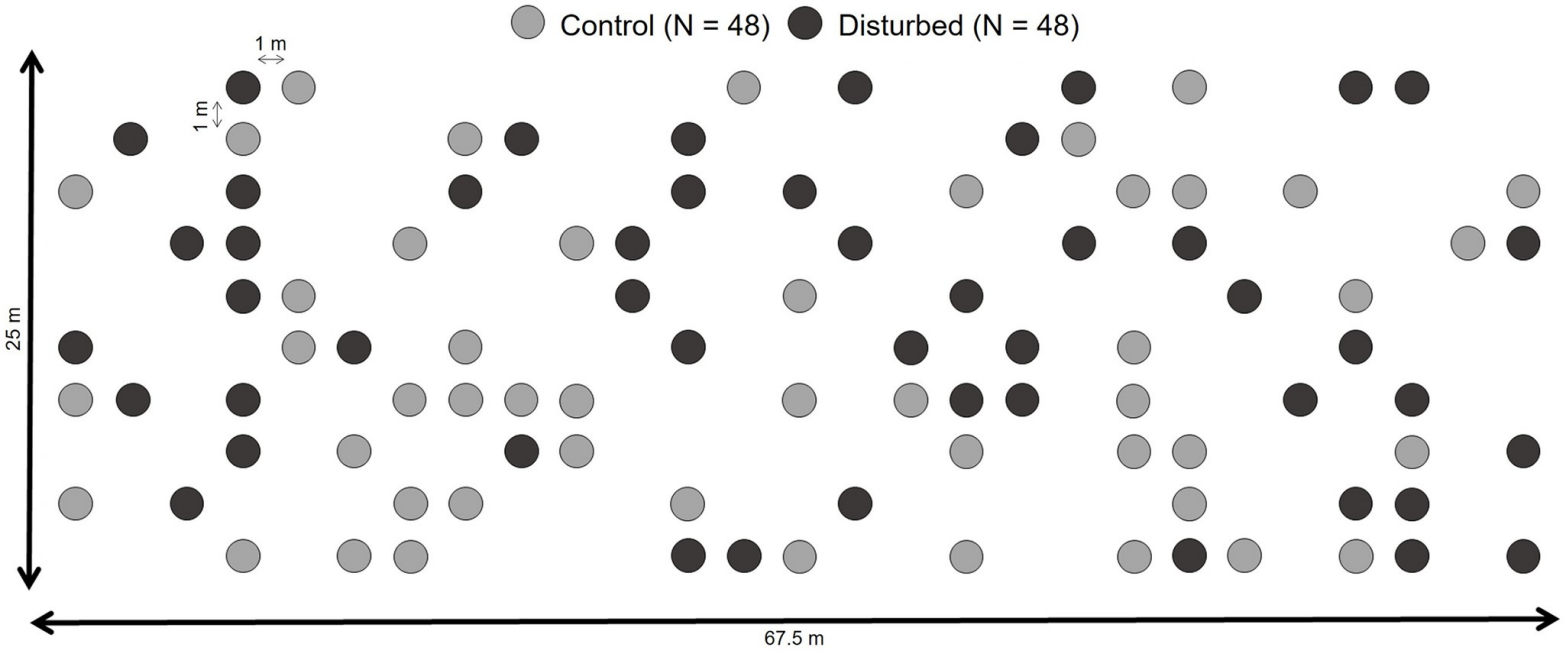
Layout of plots within the study site. The study site consists of a 67.5 m x 25 m old-field divided into 10 x 27 1 m diameter circular plots, with each plot separated by 1 m wide laneways. Forty-eight plots were randomly assigned to each treatment, control or disturbed. Light grey circles mark control plots, and dark grey circles represent disturbed plots.

Treatment plots consist of 1 m diameter circular plots organized in a grid pattern in a ~1700 m^2^ old-field (Fig 1). Rows and columns of plots are separated by 1 m laneways. Smaller plots are ideal for co-occurrence analyses in this habitat type; they have been shown to reduce the effects of abiotic variation and dispersal limitation [60,61]. The plots were established in 2009, and were randomly assigned to one of two treatments (control or disturbed), with 48 plots per treatment. The disturbance method used was the broad-spectrum herbicide, glyphosate, which inhibits plants from synthesizing certain amino acids necessary for protein structure, leading to death [62]. The glyphosate treatment was applied once, in 2009, to each disturbance plot by spraying plants within ten non-overlapping, randomly located 20 cm diameter circular areas within each 1x1 m plot. We isolated plants in these circular areas within a metal tube (~40 cm in height) to ensure the glyphosate treatment did not negatively impact surrounding plants. Glyphosate is not species-specific, and killed all plants within the areas treated. By killing plants occupying approximately 1/3 of the area in each plot, we reduced plant density and biomass in plots to below carrying capacity (reducing the role of competition) and opened up space for colonizing plants (increasing the role of dispersal).

Throughout the growing season each year from 2010 to 2012, we completed presence-absence censuses by visually surveying all plant species in each sample plot. Each year, we conducted our surveys once a month over five months (May-Sept) and then combined all survey data to capture the presence of any species found in a plot during the growing season. We measured seed mass (mg), height (m), and specific leaf area (SLA; cm^2^ g^-1^) for 20 flowering individuals per species collected from the field site (not in sample plots); we collected trait data this way to avoid disturbing experimental plots. We quantified mean seed mass, height, and SLA as the mean trait values for a plot, based on the species present in the plot. For brevity, throughout the text we refer to each trait as ‘mean *trait name*’, instead of ‘mean plot-level species *trait name*’. We also classified each plant species in the community as either stoloniferous or rhizomatous and tallied the number of stoloniferous/rhizomatous species and the number of non-stoloniferous/rhizomatous species present in each plot.

### Data analysis

We compared species richness, mean height, mean SLA, mean seed mass, and proportion of stoloniferous/rhizomatous species between control and disturbed plots across years using R.4.0.4 [63]. We fit each response variable with a linear mixed model, using plot as a random effect, with the ‘lme’ function from the package ‘nlme’ [64]. All models were fit using a normal distribution, except for species richness, which was modeled using the Poisson distribution and a log link function, and the proportion of stoloniferous/rhizomatous plants, which was modeled using the binomial distribution and a log link function. Additionally, for the binomial model, we used the number of stoloniferous/rhizomatous species as the number of successes and the number of non-stoloniferous/rhizomatous species in each plot as the number of failures. We fit models with all combinations of fixed and random predictors and used Akaike information criterion (AIC) for model selection. When multiple models were within <2 AIC units of the lowest model, we chose the model with the least number of predictors, the minimum adequate model. We used the ‘r.squaredGLMM’ function to evaluate the influence of the random effects on our response variables (package: ‘MuMIn’ [65]). We used the ‘lm’ and ‘glm’ functions [63] for models with no random predictors. We also compared species plot incidence using presence-absence data to determine how evenly species are distributed among plots in a single treatment (evenness: Evar [66]).

We calculated beta diversity to evaluate species turnover across treatments and over the three years of the experiment. We first calculated Jaccard dissimilarities using presence-absence data for species in each treatment per year using the ‘beta.pair’ function (package: ‘betapart’; [67]). Secondarily, we calculated Bray-Curtis dissimilarities from the number of plots occupied per species for each treatment and year using ‘beta.pair.abund’ (package: ‘betapart’; [67]). Both Jaccard and Bray-Curtis indices range from 0 (similar) to 1 (dissimilar).

To assess functional diversity, we calculated functional richness, evenness, divergence, and dispersion from the number of plots occupied per species for each treatment and year with mean height, mean SLA, mean seed mass, and the proportion of stoloniferous/rhizomatous species. Functional richness (F_Ric_) is a measure of the volume of functional space filled by the community, standardized from 0 (low) to 1 (high) [67]. Functional evenness (F_Eve_) is a measure of the regularity by which functional space is filled by species, considering evenness of abundances; it ranges from 0 (uneven) to 1 (even) [67]. Functional divergence (F_div_) is a measure of how abundance is distributed in functional trait space; it ranges from 0 for low divergence—when most abundant species have functional trait measures close to the centre of trait ranges, to 1 for high divergence—when most abundant species have functional trait measures at extreme ends of trait ranges [67]. Functional dispersion (F_Dis_) refers to the mean distance between each species’ measured traits and the centroid of all species in functional trait space; higher values reflect high dispersion [68]. All metrics were calculated using the ‘dbFD’ function, with a Cailliez correction (package: ‘FD’ [69]).

We used a common community-level null model test to assess co-occurrence patterns for each treatment for each of the three successive years; the performance of this test has been established through rigorous testing (e.g., [70,71]). To measure species co-occurrence, we used a common index of co-occurrence, the C-score [18,72,73]. This index quantifies the degree to which pairs of species negatively co-occur [74]. Using matrices where rows are species and columns are sites/plots, we determined the C-score for each matrix (each treatment/year combination) using the mean number of checkerboards observed across all species pairs. This metric is calculated as ∑(Si–Q)(Sj–Q) / [(R)(R– 1)/2]. Si is the sum of row i, Sj is the sum of row j, Q is the number of sites/plots where both species in a given pair are present, and R is the number of rows in the matrix [72,74]. High C-score values reflect pronounced negative co-occurrence. We compared observed community C-scores to a null distribution of C-scores for each treatment (control and disturbed). Null distributions were generated by shuffling each presence-absence matrix (one matrix for each treatment and year: 6 matrices total) and then recalculating the C-score. This process was repeated 5000 times to create a null distribution of C-scores. We shuffled the matrices using the fixed-fixed independent swap algorithm with 30,000 swaps [75]; tests using these parameters are known to have good type I and II error rates [70,76]. Co-occurrence patterns for the control and disturbance treatments were then compared using the standardized effect size (SES; e.g., [77]); Z-scores above 1.96 reflect negative co-occurrence patterns that are higher than expected under the chosen null model. We log_10_-transformed the observed C-score and the null distributions to address the positive skew of our null distributions; SES values are more validly comparable across null model tests when these distributions are normal [78]. Additionally, SES values for these tests are not confounded by species richness patterns [79]. All co-occurrence analyses were coded by T. Lavender in the Scala programming language (Scala 2.11.12).

Community-level co-occurrence tests produce a single *P*-value and a single SES value. Consequently, comparisons across treatments cannot be assessed statistically without replication across communities. It was possible, however, to examine the degree to which our SES values for each treatment were stable, using a jackknifing procedure (e.g., [19,72]). For each treatment, we removed a plot and repeated our co-occurrence analysis, recalculating the SES until we had done so for all *n*—1 combinations of plots. This approach assessed whether our co-occurrence estimates were sensitive to the removal of any single sample plot (i.e., how influential any single plot is to the SES calculated for the community). Thus, for the disturbance treatment in a given year, we conducted 48 co-occurrence null model tests, each consisting of all combinations of 47 plots. The result is essentially a measure of the reliability of the SES for the treatment/year; this method has also been used to assess the stability of phylogenetic hypotheses [80].

## Results

### Community structure across treatments

We identified 40 species growing across treatments and years in our experimental field (S1 Table). Thirty-seven of these species were found in both control and disturbed plots. Two species (*Carex vulpinoidea* and *Galium palustre*) were found only in the control treatment; one species (*C*. *cryptolepis*) was found only in the disturbed treatment. In all three cases, these species were recorded in only one plot in one year. In each year, control and disturbed treatments shared at least nine of the top ten most common species (as measured by number of plots occupied), though these species varied by year.

**Fig 2:**
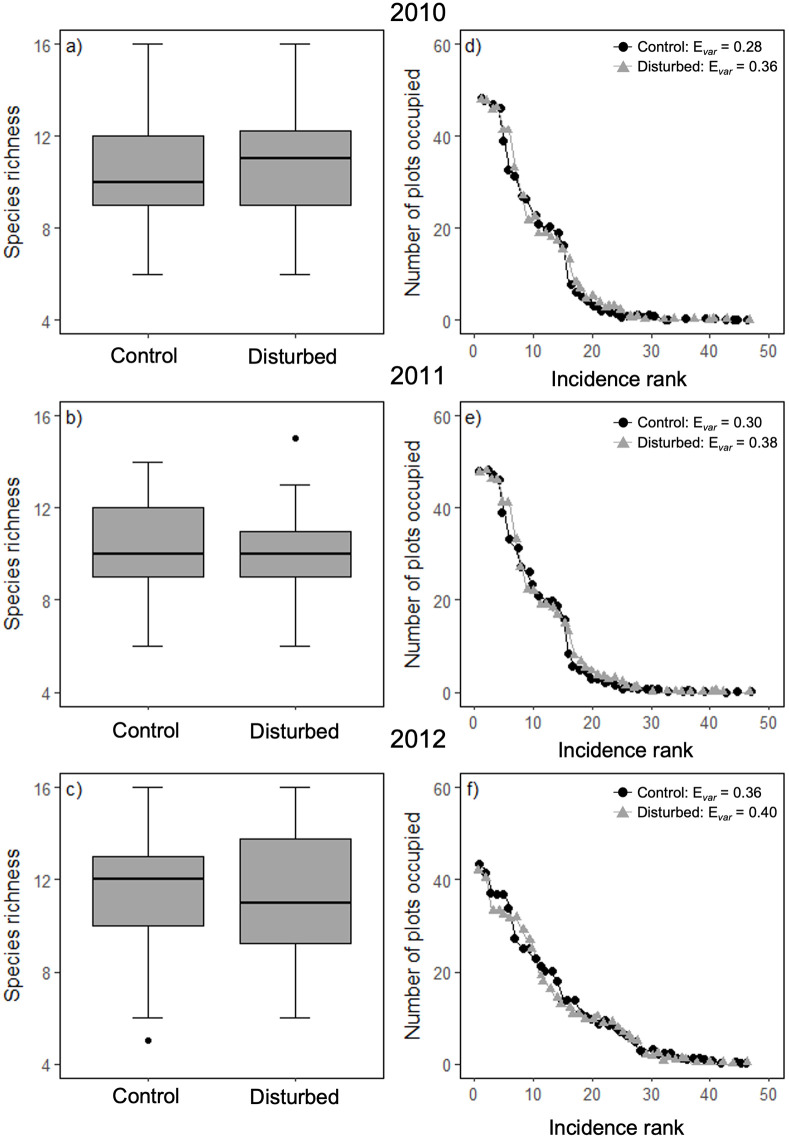
Species richness boxplots and rank incidence graph. Boxplots illustrating species richness in control and disturbed plots (48 plots per treatment) for 2010 (A), 2011 (B), and 2012 (C). Boxes represent the 25^th^ to 75^th^ percentiles of the data, and whiskers represent the 10^th^ and 90^th^ percentiles. Points above and below the box represent the 5^th^ and 95^th^ percentile outliers. Graphs showing rank incidence for the control and disturbed plots in 2010 (D), 2011 (E), and 2012 (F). Black circles represent incidence rankings for the control treatment, and grey circles represent incidence rankings for the disturbed treatment. Species richness did not differ between treatments or across years.

**Fig 3:**
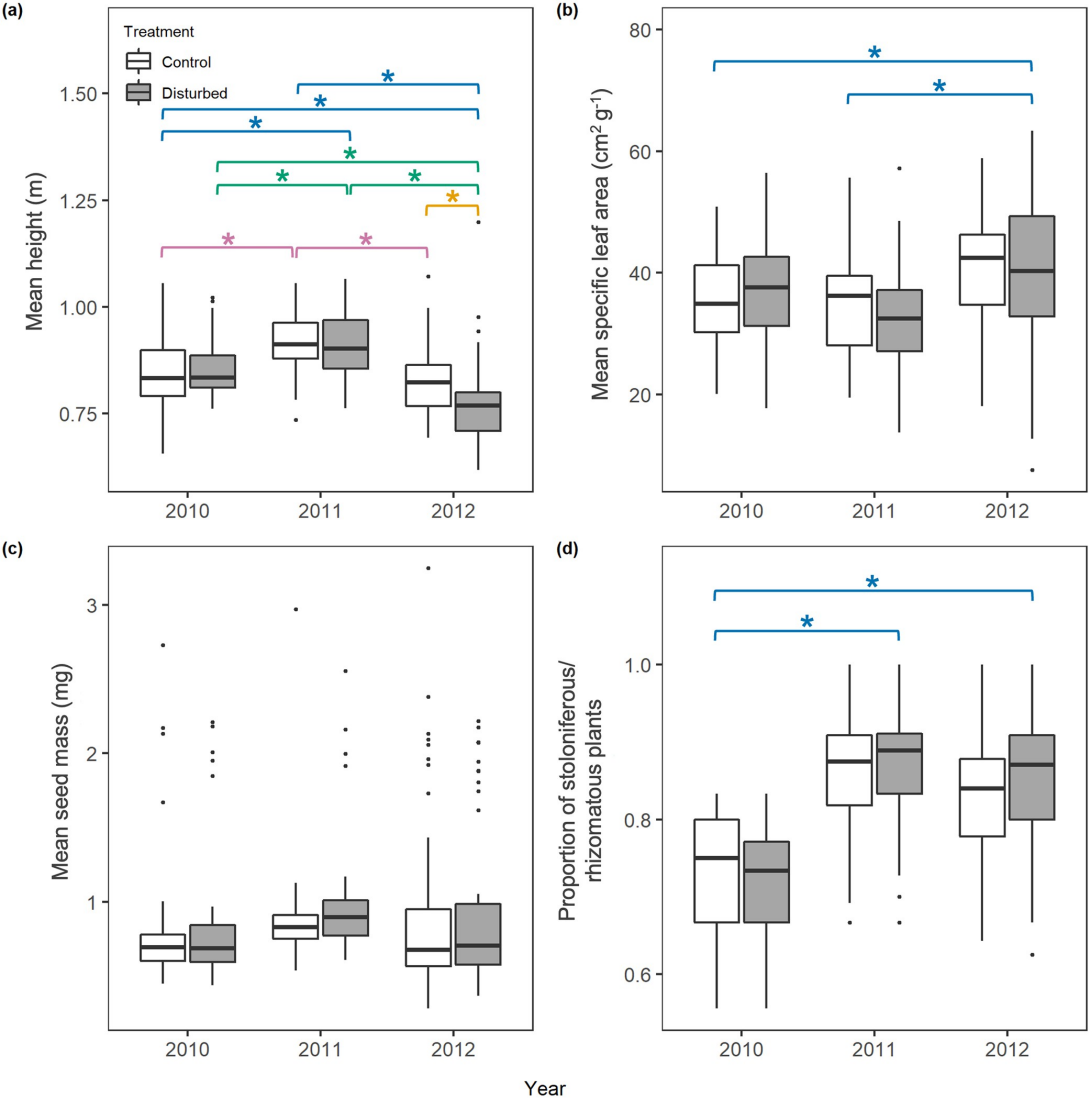
Mean height (cm), mean specific leaf area (SLA; cm^2^ g^-1^), mean seed mass (g), and proportion of stoloniferous species boxplots. Boxplots illustrating mean height (cm; A), mean SLA (cm^2^ g^-1^; B), mean seed mass (g; C), and the proportion of stoloniferous species (D) between control and disturbed treatments and across years (2010–2012). Boxes represent the 25^th^ to 75^th^ percentiles of the data, and whiskers represent the 10^th^ and 90^th^ percentiles. Points above and below the box represent the 5^th^ and 95^th^ percentile outliers. Blue significance bars marked with an asterisk indicate significant differences among years. Green significance bars marked with an asterisk indicate significant differences within the disturbed treatment among years. Pink significance bars marked with an asterisk indicate significant differences within the control treatment among years. Orange significance bars indicate significant differences between the control and disturbed treatments within a year. Mean height varied across all three years; mean height was 8% higher in 2011 than in 2012. In 2012, mean height was 5.1% lower than in 2010 and 12.1% lower than in 2011. Treatments only varied in mean height in 2012. In that year, mean height in disturbed plots was 6.4% lower. Mean SLA increased in 2012 compared to the previous two years; from 2010 to 2012, mean SLA increased by 11.4%, and from 2011 to 2012 it increased by 18.9%. Mean seed mass did not differ between treatments or years. The proportion of stoloniferous/rhizomatous species increased from 2010 to 2011 by 19.3% and from 2010 to 2012 by 14.8%, but did not differ between 2011 and 2012.

Species richness did not differ between treatments or across years (Fig 2A–2C; S2 Table). Mean height differed between treatments in 2012 only; in that year, mean height was 6.4% lower in the disturbed plots than in the control plots (Fig 3A; S3 and S4 Tables). Additionally, mean height varied across years. Plot was not retained in the final model for mean height (S2 Table). Treatments did not differ in mean SLA (Fig 3B; S5 Table). Mean SLA did not vary from 2010 to 2011, but was higher in 2012 (Fig 3B; S5 and S6 Tables). Plot identity explained 32.8% of the variation in mean SLA (S2 Table). Mean seed mass was similar between treatments and years (Fig 3C); 85.8% of the variation in mean seed mass was explained by plot identity (S2 Table). The proportion of stoloniferous/rhizomatous species did not vary between treatments in any year; however, the proportions were higher in both 2011 (+19.3%) and 2012 (+14.8%) than in 2010 (Fig 3D; S7 and S8 Tables). Plot and block were not retained in the final model (S2 Table). There was little difference in species evenness between the disturbed and control plots within and among the three treatment years (3 data points–no analysis). Evenness was similar between treatments, though it was consistently higher in disturbed plots (2010 Evar = 0.36; 2011 Evar = 0.38; 2012 Evar = 0.40) than the control plots (2010 Evar = 0.28; 2011 Evar = 0.30; 2012 Evar = 0.40) and slightly increased over the three treatment years (Fig 2D–2F).

Species composition, measured using both the presence-absence of each species within each treatment and year, and the number of plots occupied by each species within each treatment and year, was more dissimilar across years than between treatments (S9 Table). This was consistent among both dissimilarity indices. When examining species presence-absence (for each treatment/year combination: 6 matrices), Jaccard dissimilarities between pairs of years in the control treatment ranged from 0.28–0.36, and disturbed ranged from 0.2–0.4. In contrast, Jaccard dissimilarity between the control and disturbed treatments within each year ranged from 0.14–0.17. When examining the number of plots a species occupies per treatment and within each year, Bray-Curtis dissimilarities between pairs of years in the control treatment ranged from 0.29–0.49, and disturbed treatments ranged from 0.31–0.51. Bray-Curtis dissimilarities between treatments in each year ranged from 0.0058–0.081. Both indices range between 0 and 1; as such, between-treatment values were low.

Although species composition was dissimilar across years, functional diversity metrics were consistent across years (S10 Table). Functional richness was low in all treatments and years (range: 1.0 × 10^−33^–6.8 × 10^−28^). Functional evenness was higher in disturbed plots (F_eve_ = 0.74) than in control plots (F_eve_ = 0.48) in 2010; this difference diminished through time (2011: Control F_eve_ = 0.52, Disturbed F_eve_ = 0.62; 2012: Control F_eve_ = 0.59, Disturbed F_eve_ = 0.56). Functional divergence was high in both treatments and all years (range: 0.82–0.88). Finally, functional dispersion was consistently low across treatments and years (range: 0.11–0.16).

### Co-occurrence across treatments

**Fig 4:**
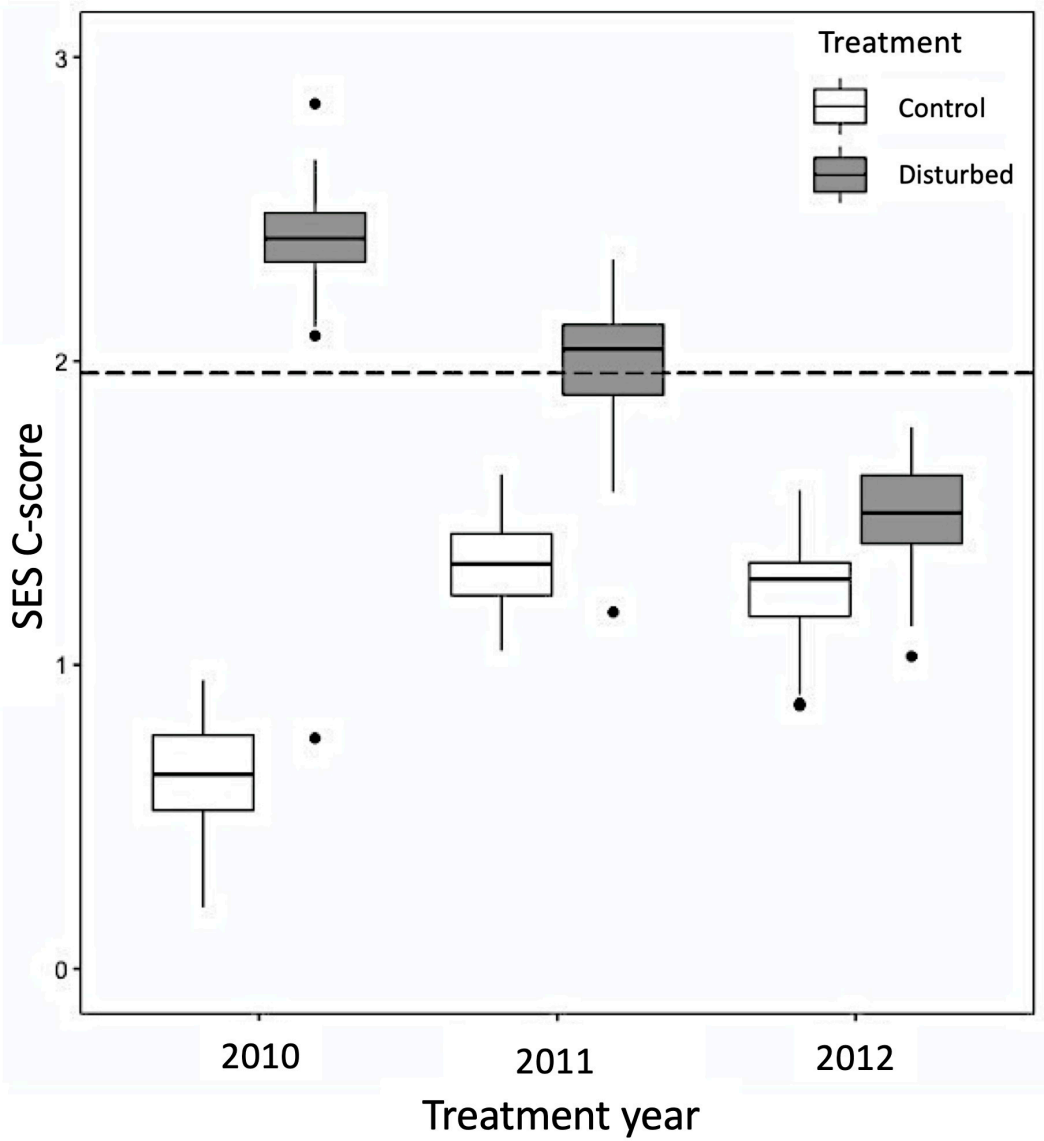
SES C-score graphs for each treatment type at the Arboretum site. Boxplots representing the median and interquartile range of the logged SES C-scores for each treatment type at the Arboretum, across treatment years. The dashed line represents a z-score of 1.96, above which negative co-occurrence patterns are generally accepted as being higher than expected by chance. The whiskers represent 95% confidence intervals for a stability estimate of the SES for the C-score. Variance within treatment and year represents SES values when each plot is removed from analysis.

The average SES of co-occurrence tests was consistently higher in disturbed plots than in control plots, indicating more pronounced negative co-occurrence relative to null expectations (Fig 4). In 2010, the difference between the SES C-score value of the control and disturbed plots was quite pronounced (Control: $\bar{\text{x}}=0.636$; Disturbed: $\bar{\text{x}}=2.349$). This difference remained throughout the three years considered; however, it diminished in subsequent years as time since the original disturbance event increased (Fig 4). Diminished differences resulted from a decline in the degree of negative co-occurrence observed for disturbed plots over time, while negative co-occurrence slightly increased in control plots over the same period.

## Discussion

Disturbance experiments/manipulations are ideal for examining the relative impact of dispersal and competition on community assembly. Mortality-inducing disturbance generates gaps for dispersal to fill, and increases the supply of resources relative to the demand, reducing competition’s importance. As such, species composition, co-occurrence patterns, and functional trait patterns (trait means and functional diversity measures) should be more heavily influenced by dispersal shortly after disturbance, with the importance of competition increasing as time passes post-disturbance [10]. We examined how co-occurrence patterns, functional trait patterns (trait means, functional trait diversity), and composition changed in the three years following disturbance to test how these patterns are influenced by dispersal and competition.

### Community structure across treatments

Mean plot-level species richness did not differ significantly between control and disturbed treatments for any of the three years considered (Fig 2A–2C). This suggests that dispersal in our community (by seed, clonal extension, etc.) ensured that any spaces opened up by disturbance-induced mortality were quickly captured, and by a similar number of species. Additionally, compositional similarity between the two treatments each year (2010: β_jac_ = 0.17, β_BC_ = 0.064; 2011: β_jac_ = 0.14, β_BC_ = 0.081; 2012: β_jac_ = 0.16, β_BC_ = 0.0058; S9 Table), and overlap in the most common species (S9 Table) suggests that gaps were largely colonized by species present within the community. Functional richness, divergence, and dispersion were similar between treatments and years. Overall, disturbed plots were more functionally even than control plots in 2010 (Disturbed 2010: 0.74; Control 2010: 0.48). Increased functional evenness indicates that species and their occurrences are more regularly distributed among plots. This difference between treatments lessened through time; functional evenness decreased in the disturbed treatment, becoming increasingly similar to the control treatment by 2012 (Disturbed 2012: 0.56; Control 2012: 0.59). Composition in both treatments in each year had low functional richness (range: 1.0 × 10^−33^–6.8 × 10^−28^; bounded by 0–1). There was also low functional dispersion in each group (range: 0.11–0.16; unbounded to an upper limit); most species trait values were similar to the mean of each trait. In contrast, functional divergence was high in all groups (range: 0.82–0.88; bounded by 0–1); very abundant species tended to have extreme trait values. Overall, treatments and time-since disturbance had limited impact on functional diversity measures.

Plant species maximum height, which several studies have linked to competitive ability [42–46,81], was not significantly higher in control plots than in disturbed plots (Fig 3A), likely because treatments were similar in species composition (S1 Table) and were dominated by many of the same species (S9 Table). Additionally, as time progressed post-disturbance, the mean height of species in disturbed plots initially increased, as might be expected if competition’s importance increased over that time, but then decreased in the third year for both treatments. There was no consistent directional trend for this functional trait. Our results are not consistent with expectations that large species should become increasingly dominant post-disturbance in a competitive environment, but are consistent with research suggesting that any size-advantage in competition is generally not realized within natural systems [82–84]. Importantly, we did not directly measure competition across the experimental time period; consequently, it is possible that competition’s importance simply did not change appreciably in the wake of our disturbance treatment. Overall, disturbance had no impact on species size in plots, and species size did not change in a consistent direction over the three years considered.

The mean seed mass of species and the proportion of species in plots that are either stoloniferous or rhizomatous changed significantly across years, but did not differ across treatments. These two functional traits did not respond to our disturbance treatment. Other factors may influence a seed’s ability to establish in gaps, such as arriving first to the area [85]; however, lottery establishment may also play a role [86]. Understanding plant communities in terms of plant functional traits remains an important goal [41]; however, it is clear from our results that the functional trait-based response of plant communities to disturbance is complex. Significant changes in mean height, mean SLA, and the proportion of stoloniferous/rhizomatous species across years, independent of treatment, resulted from changes in composition across years (S1 and S9 Tables). Research across a longer time scale is needed to determine whether these changes are driven by succession, temporal variation in soil resources, or climatic conditions.

### Co-occurrence across treatments

Co-occurrence patterns showed marked differences between the treatments. Control plots had less pronounced negative co-occurrence patterns than disturbed plots in all three years. Negative co-occurrence patterns in disturbed plots were most pronounced in the two years following disturbance (2010–2011), with patterns in 2012 approaching what was observed in control plots that year (Fig 4). Differences between treatments decreased over the three years, with negative co-occurrence in disturbed plots declining over that time. This decline in negative co-occurrence over time in disturbed plots, combined with the small increase in negative co-occurrence in control plots, highlights that disturbance can produce pronounced changes in co-occurrence patterns. The most likely mechanism for these patterns among plots in the disturbance treatment is dispersal-driven changes in composition; however, it remains possible that other factors influence these patterns. While abiotic variation among samples can contribute to co-occurrence patterns [4], the randomized location of treatment and control plots within the study field makes this an unlikely explanation [9]. Our experimental disturbance treatments may provide information about how natural disturbance from, for example, herbivores may impact co-occurrence patterns. Voles, in particular, are active and numerous in our study community, and unpublished work by our research group indicates they cause considerable plant mortality at the seedling stage. Mortality from voles may have influenced co-occurrence patterns in control plots by reducing the intensity of competition (biomass reduction, gap formation), and by limiting competitive dominance.

Our results are consistent with theoretical evidence that shows stochastic processes (such as dispersal) can contribute to negative co-occurrence patterns [15,16], as well as experimental evidence that dispersal can generate these patterns [19,72]. We propose several pathways by which increased disturbance drives patterns of negative co-occurrence. Changes to vegetation following disturbance included a slight increase in species evenness (in the number of plots occupied per species) over time within the disturbance treatment (Fig 2D–2F). Evenness was also higher in the disturbed plots compared to the control plots for all three years considered. Higher evenness in disturbed plots is related to common species in control plots being found in fewer plots after disturbance, and rarer species in control plots being found in slightly more plots in the disturbed treatment (we use ‘rare’ and ‘common’ here in reference to species that occupy very few, or very many plots respectively). Essentially, there was less dominance in disturbed plots. This may be due to a reduction in the influence of competition; however, these differences were subtle, and further research is required to be more confident in this interpretation. It is also possible that mass effects contributed to large numbers of seeds from ‘rare’ species from control plots successfully dispersing to and establishing in a higher number of disturbed plots. Additionally, unequal dispersal ability among species may have contributed to a tendency for some species to consistently be found growing apart from another. For example, species with relatively short-distance dispersal mechanisms (e.g., ballistic dispersal or gravity dispersal) would be less likely to reach areas of the field they are not immediately adjacent to, compared to species with relatively longer-distance dispersal mechanisms (e.g., wind dispersal or animal dispersal). Therefore, differences in the dispersal mode of species may limit which area of the field they can establish in, regardless of available physical space in disturbed plots; this can produce negative co-occurrence across sample plots in different parts of the field. Another explanation, which is statistical rather than biological, is that high evenness in disturbed plots in the years following disturbance increased the power of our co-occurrence tests. Recent research suggests that rare and very common species reduce the power of species pairwise co-occurrence tests; rare species are more likely to be identified as a significant negatively co-occurring species, and very common species are more likely to be identified as a significant positively co-occurring species [87]. While this has not been examined for community-wide co-occurrence tests, it is likely that a similar issue exists. It is possible then, that dispersal did not per-se contribute to more negative co-occurrence patterns, but instead allowed us to more clearly identify existing patterns. It is also possible that both explanations described above contribute to the more pronounced negative co-occurrence observed in the disturbance treatment plots in the three years immediately following disturbance.

While we cannot conclusively implicate dispersal as the driver of more pronounced negative co-occurrence in our disturbed plots relative to control plots, our results are consistent with the growing body of research demonstrating that dispersal can produce significant patterns of negative species co-occurrence (e.g., [17–19,72]). Our results conflict with the general expectation that competition-driven negative co-occurrence should increase as time passes post-disturbance. For example, along a successional gradient [32], competition should become increasingly important and consequently, negative co-occurrence should become more pronounced (e.g., [10,55]). It remains possible that competition becomes more important as time progresses after a disturbance; however, if this is the case in our study community, it does not clearly translate into stronger patterns of negative co-occurrence. It is also possible that intransitive competition, which can impact co-occurrence patterns, is a complicating element we cannot account for in our study system [10].

## Conclusions

In our study system, disturbance impacted plant community evenness, but not species richness. Disturbance also did not impact functional traits (trait means and functional diversity measures) that research has linked to competition and dispersal. The pronounced negative co-occurrence patterns observed in recently disturbed plots are consistent with recent findings that dispersal can have a substantial impact on these patterns. This research demonstrates that when disturbance leads to plant mortality, vegetation composition can change in ways that result in more pronounced negative co-occurrence. Our findings suggest that more pronounced negative co-occurrence patterns are associated with systems in which species are more evenly distributed among samples; variance in evenness may also impact the power of co-occurrence tests, influencing our results. Our results do not support an association between increased importance of competition (expected post-disturbance) and negative co-occurrence patterns, or increased plant size. Similarly, while disturbance produces gaps that plants may disperse into, seed size or clonal extension via rhizomes or stolons are not clearly important in determining which species colonize those gaps.

## Supporting information

S1 Table Summary of trait data for each recorded species as well as number of plots occupied for each species in each treatment per year. Traits include growth habit, native status, specific leaf area (cm^2^ g^-1^), seed mass (mg), height (m), and presence of stolons or rhizomes. Species are ordered by the total number of plots occupied.
(DOCX)

S2 Table Summary of final models for each response variable—Species richness, mean height, mean specific leaf area, mean seed mass, and the proportion of stoloniferous/rhizomatous species. Each model was fitted with all possible combinations of fixed predictors (treatment, year, and treatment × year) and random predictors (plot nested in block, plot) and compared using AIC scores. When multiple models fell within 2 AIC units of the lowest value, the simplest of these models with the lowest number of predictors was selected. Species richness was fitted with a generalized linear model (GLM) using the Poisson distribution and a log link function. Mean height was fitted with a linear model (LM) using the normal distribution. Both mean specific leaf area and mean seed mass were fit with linear mixed models (LMM) using the normal distribution with plot as a random variable. Proportion of stoloniferous/rhizomatous species was fitted with a generalized linear model using a binomial distribution with a log link function; the number of stoloniferous/rhizomatous species in a plot was taken as the number of successes, while the number of non-stoloniferous/rhizomatous species was taken as the number of failures. The marginal R^2^ reflects the variance explained by fixed predictors and the conditional R^2^ reflects the variance explained by both fixed and random predictors.
(DOCX)

S3 Table Mean height (cm) model results summary. Mean height was fitted with a linear model using the normal distribution. Mean height varied across years and between treatments in 2012 only.
(DOCX)

S4 Table Estimated marginal means and contrasts for mean height (cm) between treatments and years. Mean height differed across all years, and was lower in 2012 than in the previous two years. Mean height in control plots was higher in 2011 than in 2010, but decreased again by 2012 to similar heights to 2010. In disturbed plots, mean height was again higher in 2011 than 2010, but decreased between 2010 and 2012, and 2011 and 2012.
(DOCX)

S5 Table Mean specific leaf area (cm^2^ g^-1^) model results summary. Mean specific leaf area was fitted with a linear mixed model using the normal distribution, with plot as a random variable. Mean specific leaf area varied across years but did not differ between treatments.
(DOCX)

S6 Table Estimated means and contrasts for mean specific leaf area (cm^2^ g^-1^) between years. Mean specific leaf area was greater in 2012 than in both 2010 and 2011, but did not differ between 2010 and 2011.
(DOCX)

S7 Table Proportion of stoloniferous/rhizomatous species model results summary. The proportion of stoloniferous/rhizomatous species was fitted with a generalized linear model using the binomial distribution and a log link function. The number of stoloniferous/rhizomatous species in each plot was taken as the number of successes and the number of non-stoloniferous/rhizomatous species was taken as the number of failures. The proportion of stoloniferous/rhizomatous species varied across years but did not differ between treatments.
(DOCX)

S8 Table Estimated marginal means and contrasts for the proportion of stoloniferous/rhizomatous species between years. The proportion of stoloniferous/rhizomatous species was lower in 2010 than in 2011 and 2012, but did not differ between 2011 and 2012.
(DOCX)

S9 Table Jaccard (β_jac_) and Bray-Curtis (β_BC_) dissimilarities between control and disturbed treatment species pools for each year, and between each pair of years for each treatment. Jaccard dissimilarities were calculated using the recorded presence-absence of each species within each treatment per year. Bray-Curtis dissimilarities were calculated using the number of plots occupied by each species within each treatment per year. Both indices range from 0 (completely similar) to 1 (completely dissimilar).
(DOCX)

S10 Table Summary of functional diversity metrics using species’ mean height, mean specific leaf area, mean seed mass and ability to produce stolons or rhizomes as traits. Metrics include: Functional richness (F_ric_; a measure of the volume of functional space occupied by the community) functional evenness (F_eve_, a measure of the regularity that functional space is filled by species in the community, taking evenness of abundances into account) functional divergence (Fd_iv_, a measure of how abundance is distributed in functional trait space; functional divergence is high when highly abundant species have extreme trait values) and functional dispersion (F_dis_, the mean distance between species’ traits and the centroid of all species). Functional richness, evenness, and divergence range from 0 to 1, while functional dispersion is unbounded.
(DOCX)
