# Supplementary material for: Examining the relative influence of dispersal and competition on co-occurrence and functional trait patterns in response to disturbance
Source: PLoS One. 2022 Oct 7;17(10):e0275443. doi: 10.1371/journal.pone.0275443 (PMC9544017; doi:10.1371/journal.pone.0275443)
Supplement: S1 Table — Traits include growth habit, native status, specific leaf area (cm2 g-1), seed mass (mg), height (m), and presence of stolons or rhizomes. Species are ordered by the total number of plots occupied. (DOCX) [file pone.0275443.s001.docx]

**S1 Table.** Species summary

|  | Forb/ | Native/ | Maximum | Specific | Seed | Stoloniferous/ | Number of control | | | Number of disturbed | | |
| --- | --- | --- | --- | --- | --- | --- | --- | --- | --- | --- | --- | --- |
|  | graminoid | introduced | height | leaf area | mass | rhizomatous? | plots occupied | | | plots occupied | | |
|  |  |  | (m) | (cm^2^ g^-1^) | (mg) |  | 2010 | 2011 | 2012 | 2010 | 2011 | 2012 |
| *Taraxacum campylodes* G.E.Haglund | Forb | Unclear | 0.633 | 132.5 | 0.664 | Yes | 46 | 38 | 43 | 46 | 41 | 42 |
| *Ranunculus acris* L. | Forb | Unclear | 0.798 | 109.4 | 1.769 | Yes | 33 | 27 | 37 | 41 | 25 | 32 |
| *Phleum pretense* L. | Graminoid | Introduced | 1.126 | 113.8 | 0.463 | Yes | 47 | 33 | 21 | 46 | 26 | 19 |
| *Lotus corniculatus* L. | Forb | Introduced | 0.713 | 79.7 | 1.495 | No | 19 | 37 | 37 | 18 | 36 | 33 |
| *Phalaris arundinacea* L. | Forb | Native | 1.812 | 127.1 | 0.670 | Yes | 39 | 23 | 25 | 41 | 27 | 14 |
| *Agrostis gigantea* Roth | Forb | Introduced | 0.753 | 159.4 | 0.097 | Yes | 0 | 42 | 42 | 0 | 40 | 40 |
| *Symphyotrichum lanceolatum*  (Willd.) G.L.Nesom | Forb | Native | 1.076 | 157.4 | 0.163 | Yes | 20 | 26 | 37 | 19 | 25 | 33 |
| *Stellaria graminea* L. | Forb | Introduced | 0.585 | 122.6 | 0.273 | Yes | 31 | 18 | 27 | 33 | 14 | 29 |
| *Anthoxanthum odoratum* L. | Graminoid | Introduced | 0.824 | 359.5 | 0.372 | Yes | 27 | 21 | 23 | 27 | 17 | 25 |
| *Carex gracillima* Schwein. | Graminoid | Introduced | 0.744 | 87.6 | 0.325 | Yes | 2 | 48 | 10 | 2 | 48 | 9 |
| *Poa pratensis* L. | Graminoid | Native | 1.041 | 203.6 | 1.127 | No | 48 | 6 | 11 | 48 | 1 | 5 |
| *Carex pallescens* L. | Graminoid | Native | 0.653 | 183.8 | 0.938 | No | 0 | 19 | 34 | 0 | 17 | 32 |
| *Juncus tenuis* Willd. | Graminoid | Native | 0.572 | 76.2 | 0.012 | Yes | 21 | 19 | 14 | 17 | 21 | 10 |
| *Potentilla recta* L. | Forb | Introduced | 0.668 | 95.2 | 0.348 | No | 48 | 0 | 2 | 48 | 0 | 1 |
| *Bromus inermis* Leyss. | Graminoid | Unclear | 1.565 | 96.9 | 2.900 | Yes | 1 | 48 | 0 | 0 | 48 | 1 |
| *Euthamia graminifolia* (L.) Nutt. | Forb | Native | 0.94 | 142.7 | 0.061 | Yes | 6 | 17 | 14 | 15 | 12 | 11 |
| *Veronica serpyllifolia* L. | Forb | Introduced | 0.154 | 129.3 | 0.060 | Yes | 0 | 9 | 25 | 0 | 7 | 32 |
| *Plantago lanceolata* L. | Forb | Introduced | 0.77 | 94.9 | 1.697 | Yes | 1 | 17 | 14 | 0 | 19 | 12 |
| *Cerastium glomeratum* Thuill. | Forb | Introduced | 0.666 | 183.2 | 0.130 | Yes | 23 | 0 | 8 | 22 | 1 | 8 |
| *Juncus effuses* L. | Graminoid | Native | 1.075 | — | 0.010 | Yes | 1 | 17 | 18 | 1 | 11 | 13 |
| *Plantago major* L. | Forb | Introduced | 0.202 | 46.1 | 0.367 | Yes | 26 | 3 | 1 | 22 | 1 | 1 |
| *Leucanthemum vulgare* (Vaill.) Lam. | Forb | Introduced | 0.717 | 199.9 | 0.517 | Yes | 1 | 3 | 9 | 1 | 8 | 27 |
| *Fragaria virginiana* Mill. | Forb | Native | 0.255 | 106.8 | 0.489 | Yes | 20 | 1 | 0 | 13 | 3 | 6 |
| *Trifolium pretense* L. | Forb | Introduced | 0.535 | 120.2 | 1.605 | No | 16 | 1 | 2 | 19 | 3 | 2 |
| *Festuca rubra* L. | Graminoid | Introduced | 1.208 | 112.9 | 1.015 | Yes | 0 | 0 | 20 | 0 | 0 | 18 |
| *Vicia cracca* L. | Forb | Introduced | 0.887 | 14.8 | 17.784 | Yes | 5 | 1 | 8 | 5 | 4 | 10 |
| *Prunella vulgaris* L. | Forb | Introduced | 0.387 | 105.4 | 0.968 | Yes | 4 | 0 | 3 | 8 | 4 | 11 |
| *Solidago canadensis* L. | Forb | Native | 1.072 | 146.3 | 0.078 | Yes | 3 | 4 | 6 | 7 | 5 | 5 |
| *Pilosella aurantiaca* (L.) F.W.Schultz & Sch.Bip. | Forb | Introduced | 0.534 | 160.5 | 0.157 | Yes | 0 | 0 | 10 | 3 | 0 | 16 |
| *Equisetum arvense* L. | Forb | Native | 0.504 | — | — | Yes | 2 | 1 | 7 | 0 | 4 | 10 |
| *Elymus repens* (L.) Gould | Graminoid | Introduced | 0.98 | 104.8 | 3.673 | Yes | 0 | 0 | 9 | 0 | 0 | 7 |
| *Scirpus atrovirens* Willd. | Graminoid | Native | 1.307 | 57.3 | 0.063 | No | 1 | 1 | 5 | 0 | 0 | 2 |
| *Daucus carota* L. | Forb | Introduced | 1.29 | — | 0.759 | No | 2 | 0 | 0 | 5 | 0 | 0 |
| *Sisyrinchium montanum* Greene | Forb | Introduced | 0.175 | 83.3 | 0.627 | Yes | 0 | 0 | 3 | 0 | 0 | 2 |
| *Trifolium repens* L. | Forb | Native | 0.417 | 141.1 | 1.003 | Yes | 1 | 0 | 3 | 1 | 0 | 0 |
| *Agrimonia gryposepala* Wallr. | Forb | Native | 1.5 | — | 4.380 | Yes | 1 | 0 | 0 | 3 | 0 | 0 |
| *Cirsium arvense* (L.) Scop. | Forb | Introduced | 0.8 | 118.7 | 1.097 | No | 0 | 0 | 1 | 0 | 1 | 1 |
| *Carex cryptolepis* Mack. | Graminoid | Native | 0.654 | 142.9 | 0.538 | No | 0 | 0 | 0 | 0 | 0 | 1 |
| *Carex vulpinoidea* Michx. | Forb | Native | 0.788 | 208.6 | 0.328 | No | 0 | 0 | 1 | 0 | 0 | 0 |
| *Galium palustre* L. | Forb | Native | 0.6 | — | 0.910 | Yes | 0 | 0 | 1 | 0 | 0 | 0 |
