# Supplementary material for: Examining the relative influence of dispersal and competition on co-occurrence and functional trait patterns in response to disturbance
Source: PLoS One. 2022 Oct 7;17(10):e0275443. doi: 10.1371/journal.pone.0275443 (PMC9544017; doi:10.1371/journal.pone.0275443)
Supplement: S2 Table — Each model was fitted with all possible combinations of fixed predictors (treatment, year, and treatment × year) and random predictors (plot nested in block, plot) and compared using AIC scores. When multiple models fell within 2 AIC units of the lowest value, the simplest of these models with the lowest number of predictors was selected. Species richness was fitted with a generalized linear model (GLM) using the Poisson distribution and a log link function. Mean height was fitted with a linear model (LM) using the normal distribution. Both mean specific leaf area and mean seed mass were fit with linear mixed models (LMM) using the normal distribution with plot as a random variable. Proportion of stoloniferous/rhizomatous species was fitted with a generalized linear model using a binomial distribution with a log link function; the number of stoloniferous/rhizomatous species in a plot was taken as the number of successes, while the number of non-stoloniferous/rhizomatous species was taken as the number of failures. The marginal R2 reflects the variance explained by fixed predictors and the conditional R2 reflects the variance explained by both fixed and random predictors. (DOCX) [file pone.0275443.s002.docx]

**S2 Table.** Final model summary

| Response variable | Model type | Distribution | Fixed predictors | Significant fixed predictors | Random predictors | Marginal  R^2^ | Conditional R^2^ |
| --- | --- | --- | --- | --- | --- | --- | --- |
| Species richness | GLM | Poisson | -- | -- | -- | -- | -- |
| Mean height | LM | Normal | Treatment, Year,  Treatment × Year | Year,  Treatment × Year | -- | 0.27 | -- |
| Mean specific leaf area | LMM | Normal | Year | Year | Plot | 0.072 | 0.40 |
| Mean seed mass | LMM | Normal | -- | -- | Plot | 0 | 0.86 |
| Proportion of stoloniferous/  rhizomatous species | GLM | Binomial | Year | Year | -- | -- | -- |
