# Supplementary material for: Examining the relative influence of dispersal and competition on co-occurrence and functional trait patterns in response to disturbance
Source: PLoS One. 2022 Oct 7;17(10):e0275443. doi: 10.1371/journal.pone.0275443 (PMC9544017; doi:10.1371/journal.pone.0275443)
Supplement: S3 Table — Mean height was fitted with a linear model using the normal distribution. Mean height varied across years and between treatments in 2012 only. (DOCX) [file pone.0275443.s003.docx]

**S3 Table.** Mean height model results summary

|  | χ^2^ | df | *P* |
| --- | --- | --- | --- |
| Treatment | 1.6922 | 1 | 0.19 |
| Year | 46.385 | 2 | <0.001* |
| Treatment:Year | 7.2411 | 2 | 0.03* |
