# Supplementary material for: Examining the relative influence of dispersal and competition on co-occurrence and functional trait patterns in response to disturbance
Source: PLoS One. 2022 Oct 7;17(10):e0275443. doi: 10.1371/journal.pone.0275443 (PMC9544017; doi:10.1371/journal.pone.0275443)
Supplement: S4 Table — Mean height differed across all years, and was lower in 2012 than in the previous two years. Mean height in control plots was higher in 2011 than in 2010, but decreased again by 2012 to similar heights to 2010. In disturbed plots, mean height was again higher in 2011 than 2010, but decreased between 2010 and 2012, and 2011 and 2012. (DOCX) [file pone.0275443.s004.docx]

**S4 Table.** Mean height contrasts by year and by treatment–year

| Year | Estimated marginal mean (m) | Year | Estimated marginal mean (m) | SE | df | t ratio | *P* | Percent change (%) |
| --- | --- | --- | --- | --- | --- | --- | --- | --- |
| 2010 | 0.846 | 2011 | 0.914 | 0.0117 | 282 | -5.836 | <0.001* | 8.0 |
| 2010 | 0.846 | 2012 | 0.803 | 0.0117 | 282 | 3.727 | <0.001* | -5.1 |
| 2011 | 0.914 | 2012 | 0.803 | 0.0117 | 282 | 9.563 | <0.001* | -12.1 |

| Treatment | Year | Estimated marginal mean (m) | Treatment | Year | Estimated marginal mean (m) | SE | df | t ratio | *P* | Percent change (%) |
| --- | --- | --- | --- | --- | --- | --- | --- | --- | --- | --- |
| Control | 2010 | 0.837 | Disturbed | 2010 | 0.855 | 0.0165 | 282 | -1.067 | 0.89 | 2.2 |
| Control | 2011 | 0.915 | Disturbed | 2011 | 0.914 | 0.0165 | 282 | 0.098 | 1 | -0.1 |
| Control | 2012 | 0.829 | Disturbed | 2012 | 0.776 | 0.0165 | 282 | 3.147 | 0.022* | -6.4 |
| Control | 2010 | 0.837 | Control | 2011 | 0.915 | 0.0165 | 282 | -4.709 | <0.001* | 9.3 |
| Control | 2010 | 0.837 | Control | 2012 | 0.829 | 0.0165 | 282 | 0.529 | 0.995 | -1.0 |
| Control | 2011 | 0.915 | Control | 2012 | 0.829 | 0.0165 | 282 | 5.238 | <0.001* | -9.4 |
| Disturbed | 2010 | 0.855 | Disturbed | 2011 | 0.914 | 0.0165 | 282 | -3.544 | 0.0061* | 6.9 |
| Disturbed | 2010 | 0.855 | Disturbed | 2012 | 0.776 | 0.0165 | 282 | 4.742 | <0.001* | -9.2 |
| Disturbed | 2011 | 0.914 | Disturbed | 2012 | 0.776 | 0.0165 | 282 | 8.287 | <0.001* | -15.1 |
