# Supplementary material for: Examining the relative influence of dispersal and competition on co-occurrence and functional trait patterns in response to disturbance
Source: PLoS One. 2022 Oct 7;17(10):e0275443. doi: 10.1371/journal.pone.0275443 (PMC9544017; doi:10.1371/journal.pone.0275443)
Supplement: S5 Table — Mean specific leaf area was fitted with a linear mixed model using the normal distribution, with plot as a random variable. Mean specific leaf area varied across years but did not differ between treatments. (DOCX) [file pone.0275443.s005.docx]

**S5 Table.** SLA model results summary

|  | χ^2^ | df | *P* |
| --- | --- | --- | --- |
| Year | 34.676 | 2 | <0.001* |
