# Supplementary material for: Examining the relative influence of dispersal and competition on co-occurrence and functional trait patterns in response to disturbance
Source: PLoS One. 2022 Oct 7;17(10):e0275443. doi: 10.1371/journal.pone.0275443 (PMC9544017; doi:10.1371/journal.pone.0275443)
Supplement: S6 Table — Mean specific leaf area was greater in 2012 than in both 2010 and 2011, but did not differ between 2010 and 2011. (DOCX) [file pone.0275443.s006.docx]

**S6 Table.** SLA contrasts across years

| Year | Estimated marginal mean (cm^2^ g^-1^) | Year | Estimated marginal mean (cm^2^ g^-1^) | SE | df | t ratio | *P* | Percent change (%) |
| --- | --- | --- | --- | --- | --- | --- | --- | --- |
| 2010 | 36.1 | 2011 | 33.8 | 1.1 | 190 | 2.123 | 0.088 | -6.4 |
| 2010 | 36.1 | 2012 | 40.2 | 1.1 | 190 | -3.695 | <0.001* | 11.4 |
| 2011 | 33.8 | 2012 | 40.2 | 1.1 | 190 | -5.818 | <0.001* | 18.9 |
