# Supplementary material for: Examining the relative influence of dispersal and competition on co-occurrence and functional trait patterns in response to disturbance
Source: PLoS One. 2022 Oct 7;17(10):e0275443. doi: 10.1371/journal.pone.0275443 (PMC9544017; doi:10.1371/journal.pone.0275443)
Supplement: S7 Table — The proportion of stoloniferous/rhizomatous species was fitted with a generalized linear model using the binomial distribution and a log link function. The number of stoloniferous/rhizomatous species in each plot was taken as the number of successes and the number of non-stoloniferous/rhizomatous species was taken as the number of failures. The proportion of stoloniferous/rhizomatous species varied across years but did not differ between treatments. (DOCX) [file pone.0275443.s007.docx]

**S7 Table.** Proportion of stoloniferous/rhizomatous model results summary

|  | Likelihood-ratio χ^2^ | df | *P* |
| --- | --- | --- | --- |
| Year | 69.684 | 2 | <0.001* |
