# Supplementary material for: Examining the relative influence of dispersal and competition on co-occurrence and functional trait patterns in response to disturbance
Source: PLoS One. 2022 Oct 7;17(10):e0275443. doi: 10.1371/journal.pone.0275443 (PMC9544017; doi:10.1371/journal.pone.0275443)
Supplement: S8 Table — The proportion of stoloniferous/rhizomatous species was lower in 2010 than in 2011 and 2012, but did not differ between 2011 and 2012. (DOCX) [file pone.0275443.s008.docx]

**S8 Table.** Proportion of stoloniferous/rhizomatous contrasts across years

| Year | Estimated marginal mean | Year | Estimated marginal mean | SE | z ratio | *P* | Percent change (%) |
| --- | --- | --- | --- | --- | --- | --- | --- |
| 2010 | 0.73 | 2011 | 0.87 | 0.12 | -7.664 | <0.001* | 19.3 |
| 2010 | 0.73 | 2012 | 0.84 | 0.11 | -5.943 | <0.001* | 14.8 |
| 2011 | 0.87 | 2012 | 0.84 | 0.128 | 2.095 | 0.091 | -3.8 |
