# Supplementary material for: Examining the relative influence of dispersal and competition on co-occurrence and functional trait patterns in response to disturbance
Source: PLoS One. 2022 Oct 7;17(10):e0275443. doi: 10.1371/journal.pone.0275443 (PMC9544017; doi:10.1371/journal.pone.0275443)
Supplement: S9 Table — Jaccard dissimilarities were calculated using the recorded presence-absence of each species within each treatment per year. Bray-Curtis dissimilarities were calculated using the number of plots occupied by each species within each treatment per year. Both indices range from 0 (completely similar) to 1 (completely dissimilar). (DOCX) [file pone.0275443.s009.docx]

**S9 Table.** Dissimilarity summary

|  |  |  | Jaccard | Bray-Curtis |
| --- | --- | --- | --- | --- |
|  | Group 1 | Group 2 | dissimilarity (β_jac_) | dissimilarity (β_BC_) |
| *Between treatments - within years* | | |  |  |
| Year |  |  |  |  |
| 2010 | Control | Disturbed | 0.17 | 0.064 |
| 2011 | Control | Disturbed | 0.14 | 0.081 |
| 2012 | Control | Disturbed | 0.16 | 0.0058 |
|  |  |  |  |  |
| *Within treatments - between years* | | |  |  |
| Treatment |  |  |  |  |
| Control | 2010 | 2011 | 0.28 | 0.49 |
| Control | 2010 | 2012 | 0.36 | 0.49 |
| Control | 2011 | 2012 | 0.35 | 0.29 |
| Disturbed | 2010 | 2011 | 0.36 | 0.51 |
| Disturbed | 2010 | 2012 | 0.40 | 0.48 |
| Disturbed | 2011 | 2012 | 0.20 | 0.31 |
