# Supplementary material for: Examining the relative influence of dispersal and competition on co-occurrence and functional trait patterns in response to disturbance
Source: PLoS One. 2022 Oct 7;17(10):e0275443. doi: 10.1371/journal.pone.0275443 (PMC9544017; doi:10.1371/journal.pone.0275443)
Supplement: S10 Table — Metrics include: Functional richness (Fric; a measure of the volume of functional space occupied by the community) functional evenness (Feve, a measure of the regularity that functional space is filled by species in the community, taking evenness of abundances into account) functional divergence (Fdiv, a measure of how abundance is distributed in functional trait space; functional divergence is high when highly abundant species have extreme trait values) and functional dispersion (Fdis, the mean distance between species’ traits and the centroid of all species). Functional richness, evenness, and divergence range from 0 to 1, while functional dispersion is unbounded. (DOCX) [file pone.0275443.s010.docx]

**Table S10.** Summary of functional diversity metrics for each treatment across years

|  | 2010 | | 2011 | | 2012 | |
| --- | --- | --- | --- | --- | --- | --- |
|  | Control | Disturbed | Control | Disturbed | Control | Disturbed |
| Functional richness (F_Ric_) | 4.1 × 10^-31^ | 1.0 × 10^-33^ | 1.0 × 10^-33^ | 5.5 × 10^-32^ | 4.9 × 10^-28^ | 6.8 × 10^-28^ |
| Functional evenness (F_Eve_) | 0.48 | 0.74 | 0.52 | 0.62 | 0.59 | 0.56 |
| Functional divergence (F_Div_) | 0.88 | 0.88 | 0.82 | 0.83 | 0.85 | 0.83 |
| Functional dispersion (F_Dis_) | 0.16 | 0.16 | 0.11 | 0.11 | 0.13 | 0.12 |

**Functional richness:** species are mapped in trait space---richness is quantified from the volume of the minimum convex hull (smallest area formed by the outer points). Higher volume = larger range of traits. Original metric has no upper limit; can be standardized from 0-1

**Functional evenness:** species are mapped in trait space and linked via the minimum spanning tree (links points via shortest total distance). Quantifies how even branch lengths and abundance (if given) are. Ranges from 0 (uneven) to 1 (even)

**Functional divergence:** species are mapped in trait space; 'centre of gravity' is calculated at the centre of occupied volume (minimum convex hull). Deviation from mean distance to the centre is calculated foreach species. Divergence is low when most abundant species have traits close to the centre of gravity (0); divergence is high when most abundant species have traits far from the centre of gravity (1).

**Functional dispersion:** species are mapped in trait space; dispersion refers to the mean distance of all individual species to the weighted (if given abundance data) centroid (not centre of gravity) of all species. Not constrained to an upper limit (as far as I can tell; plot of simulated values in Laliberte & Legendre 2010 goes to 2.5)
